# Joint analysis of duration of ventilation, length of intensive care, and mortality of COVID-19 patients: a multistate approach

**DOI:** 10.1186/s12874-020-01082-z

**Published:** 2020-08-11

**Authors:** Derek Hazard, Klaus Kaier, Maja von Cube, Marlon Grodd, Lars Bugiera, Jerome Lambert, Martin Wolkewitz

**Affiliations:** 1 Institute of Medical Biometry and Statistics, Faculty of Medicine and Medical Center - University of Freiburg, Stefan-Meier-Str. 26, 79104 Freiburg, Germany; 2 Freiburg Center for Data Analysis and Modeling- University of Freiburg, Ernst-Zermelo-Str. 1, 79104 Freiburg, Germany; 3 INSERM U1153 CRESS, Epidemiology and Clinical Statistics for Tumor, Respiratory, and Resuscitation Assessments (ECSTRRA) Team, Hôpital Saint Louis, 1 av Claude Vellefaux, 75010 Paris, France

**Keywords:** SARS-CoV-2, Multistate model, Length of stay, Competing risks, Mechanical ventilation

## Abstract

**Background:**

The clinical progress of patients hospitalized due to COVID-19 is often associated with severe pneumonia which may require intensive care, invasive ventilation, or extracorporeal membrane oxygenation (ECMO). The length of intensive care and the duration of these supportive therapies are clinically relevant outcomes. From the statistical perspective, these quantities are challenging to estimate due to episodes being time-dependent and potentially multiple, as well as being determined by the competing, terminal events of discharge alive and death.

**Methods:**

We used multistate models to study COVID-19 patients’ time-dependent progress and provide a statistical framework to estimate hazard rates and transition probabilities. These estimates can then be used to quantify average sojourn times of clinically important states such as intensive care and invasive ventilation. We have made two real data sets of COVID-19 patients (*n* = 24* and *n* = 53**) and the corresponding statistical code publically available.

**Results:**

The expected lengths of intensive care unit (ICU) stay at day 28 for the two cohorts were 15.05* and 19.62** days, while expected durations of mechanical ventilation were 7.97* and 9.85** days. Predicted mortality stood at 51%* and 15%**. Patients mechanically ventilated at the start of the example studies had a longer expected duration of ventilation (12.25*, 14.57** days) compared to patients non-ventilated (4.34*, 1.41** days) after 28 days. Furthermore, initially ventilated patients had a higher risk of death (54%* and 20%** vs. 48%* and 6%**) after 4 weeks. These results are further illustrated in stacked probability plots for the two groups from time zero, as well as for the entire cohort which depicts the predicted proportions of the patients in each state over follow-up.

**Conclusions:**

The multistate approach gives important insights into the progress of COVID-19 patients in terms of ventilation duration, length of ICU stay, and mortality. In addition to avoiding frequent pitfalls in survival analysis, the methodology enables active cases to be analyzed by allowing for censoring. The stacked probability plots provide extensive information in a concise manner that can be easily conveyed to decision makers regarding healthcare capacities. Furthermore, clear comparisons can be made among different baseline characteristics.

## Background

Most individuals infected with SARS-CoV-2 will experience mild or moderate symptoms (such as cough, fever, shortness of breath) and do not need hospitalization. In contrast, those with a severe pneumonia require clinical support.

The temporal dynamics of illness severity among hospitalized Covid-19 patients can be described in terms of length of stay in the intensive care unit, duration of invasive ventilation, and the probability of death. The present paper demonstrates the application of statistical methods for analyzing these time-dependent types of data from hospitalized Covid-19 patients. These models effectively map the progression of diseased patients during their ICU stay. The methodology also avoids common pitfalls and biases that arise during the analysis of hospital data when using less sophisticated survival analysis methods [1, 2]. For example, treating exposures as time-fixed at baseline that can, in fact, vary over time leads to the time-dependent bias [3, 4]. Furthermore, ignoring competing events that can impede the observation of the event of interest can introduce bias and facilitate false predictions [5]. The ability of multistate models to avoid these biases was demonstrated in analyzing the effect of treatment with Oseltamivir (Tamiflu) on hospital mortality and length of stay in confirmed pandemic influenza A/H1N1 2009 infected patients [6, 7]. In this current demonstration, invasive mechanical ventilation is treated as time-dependent while competing risks are adequately accounted for, thus avoiding the aforementioned biases.

For illustration of the methods used, we used two real data examples extracted from figures in The New England Journal of Medicine. The first one was a case series of 24 laboratory-verified COVID-19 intensive care patients admitted to hospital ICUs in the Seattle area [8]. The second one was a sample of 53 COVID-19 patients from North America, Europe, and Japan that we extracted from a figure depicting a recent study of patients treated with compassionate-use Remdesivir [9]. It should be noted that the two data sets are used for demonstration and not for comparing the two cohorts. For both data sets we estimate duration in the ICU and under mechanical ventilation using multistate model methodology.

## Methods

**Fig. 1:**
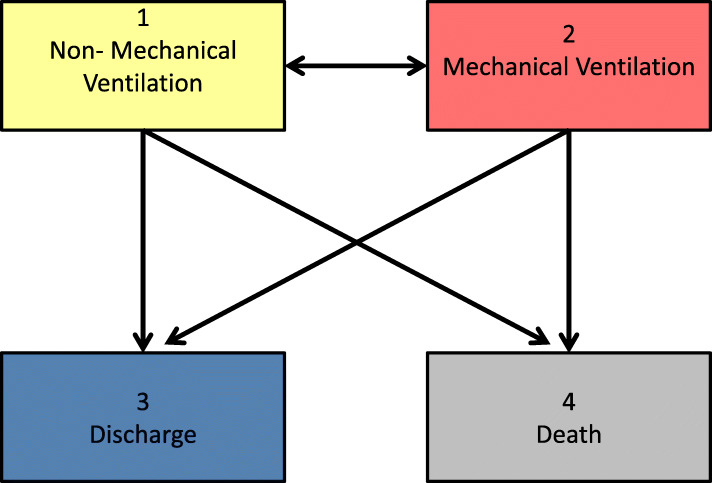
Multistate Model. Multistate model for patients admitted to the ICU with severe COVID-19. The boxes represent potential states for an ICU patient. The arrows represent the potential transitions among the states

Multistate models are a powerful tool to study the course of ICU stay of diseased patients. COVID-19 observational studies can, for example, be analyzed with the model shown in Fig. 1. In this model, patients may enter the study in one of two initial states: State 1: ICU without invasive mechanical ventilation (“Non-MV”) and State 2: ICU with mechanical ventilation (“MV). These two states are called transient states. The model includes two absorbing states from which a patient no longer transitions: discharged alive from the ICU (State 3: “Discharge”) and dead (State 4: “Death”). From ICU without ventilation, a patient can either be ventilated, discharged, or die. From ventilation, a patient can transition into non-ventilation, discharge, or death. Patients can repeatedly transition between ventilation and non-ventilation.

### Estimands

Formally the course of a patients ICU stay is described with a time-inhomogeneous Markov chain given by {*X*(*t*), *t* ≥ 0} with finite state space *S* = {1, 2, 3, 4} and follow-up time *τ*. *X*(*t*) denotes the state occupied at time t. Various estimands are of interest. First, we define the probability to move from one state to another within the multistate model. This includes, for example, the ICU mortality risk and the discharge probability.

The (Markovian) transition probabilities are.

1 $$ {P}_{lm}\left(s,t\right)=P\left(X(t)=m\left|X(s)=l\right.\right),\kern0.5em \mathrm{with}\kern0.5em l,m\kern0.5em \in S,l\ne m\kern0.5em \mathrm{and}\kern0.5em 0\le s<t\le \tau $$

and interpreted as the probabilities to transition from State l, occupied at time *s*, to State *m* within the time interval (*s*, *t*]. The Markov property states that this probability depends only on the current time *s* and the current state occupied at *s*, but not on past events. Using formula (1) we can define the transition hazards as

2 $$ {\alpha}_{lm}(t)={\lim}_{\Delta t\to 0}\frac{P_{lm}\left(t,t+\Delta \right)}{\Delta t} $$

The transition hazards are represented graphically by the arrows between states in Fig. 1.

Subsequently, we can define cause specific cumulative hazards as

3 $$ {A}_{lm}(t)={\int}_0^t{\alpha}_{lm}(u) du $$

For more details, we refer to [10–12].

We analyze the course of a patients hospital stay from study entry (*s* = 0) in the following. The probabilities to start either in State 1 or State 2 define the initial distribution, which is given by.

4 $$ P\left(X(0)=1\right)\ \mathrm{and}\ P\left(X(0)=2\right) $$

The state occupation probabilities are

5 $$ {P}_1(t)={P}_{11}(t)\cdotp P\left(X(0)=1\right)+{P}_{21}(t)\cdotp P\left(X(0)=2\right) $$

and

6 $$ {P}_2(t)={P}_{12}(t)\cdotp P\left(X(0)=1\right)+{P}_{22}(t)\cdotp P\left(X(0)=2\right) $$

To determine estimands for the full cohort, we could take into account the initial distribution and use (5) and (6) in the equations that follow. However, to focus on patients that start in a specific state, (5) reduces to

7 $$ {P}_1(t)=P\left(X(t)=1\right) $$

for patients that start in State 1 and (6) reduces to

8 $$ {P}_2(t)=P\left(X(t)=2\right) $$

for patients that start in State 2. We will use (7) and (8) in what follows.

The state occupation probabilities can be used to derive the length of stay in the ICU and the duration of mechanical ventilation. The sojourn time spent in the ICU non-ventilated (truncated after, for example, 28 days) is formally given by

9 $$ {E}_{Non- MV, Non- MV}^{\tau =28}={\int}_0^{28}P\left(X(u)=1\right) du, $$

if the patient started in State 1 and

10 $$ {E}_{MV, Non- MV}^{\tau =28}={\int}_0^{28}P\left(X(u)=1|X(0)=2\right) du, $$

if the patient started in State 2.

Similarly, the duration of MV is given by

11 $$ {E}_{Non- MV, MV}^{\tau =28}={\int}_0^{28}P\left(X(u)=2|X(0)=1\right) du, $$

if the patient started in State 1 and

12 $$ {E}_{MV, MV}^{\tau =28}={\int}_0^{28}P\left(X(u)=2\right) du, $$

if the patient started in State 2.

The total length of stay in the ICU (irrespective of being ventilated or not) up to a maximum of 28 days is simply the sum

13 $$ {E}_{Non- MV, Non- MV}^{\tau =28}+{E}_{Non- MV, MV}^{\tau =28} $$

if the patient started in State 1 and

14 $$ {E}_{MV, Non- MV}^{\tau =28}+{E}_{MV, MV}^{\tau =28} $$

if the patient started in State 2.

We have limited *S* to the 4 states depicted in Fig. 1. However, *S* can take the value of a finite number of *J* states and the methodology still holds. For an example of an extended model, see Additional file 11.

### Estimators

We used the R package *mstate* to estimate the transition and state occupation probabilities for the patients over the course of their ICU stay up to 28 days, implying administrative censoring at day 28. The *mstate* package employs Aalen-Johansen estimators which are implemented within the R-function *probtrans.* The probabilities can be estimated from both initial states (ventilated (State 2) and non-ventilated (State 1) admission to the ICU).

The Aalen-Johansen estimators are based on matrix multiplication and therefore depend fundamentally on the Markov assumption. As described in detail by Allignol et al. [13] and Beyersmann et al. [14], estimation is based on the cause-specific cumulative hazards indicated in formula (3). These are informally given by

15 $$ {\hat{A}}_{lm}(t)=\sum \limits_{k=1}^L\frac{number\ of\ observed\ l\to m\  transitions\  at\ {t}_k}{number\ of\ in dividuals\  at\  risk\ in\ state\ l\  just\ prior\ to\ {t}_k} $$

where *L* is the total number of events up to time t and *t*_*k*_, *k* = 1, …*L*, are the event times. Then, using a representation of the state occupation probabilities as product integral (explained in detail in Additional file 12), we have

16 $$ \hat{\boldsymbol{P}}\left(0,t\right)=\prod \limits_{k=1}^L\left(\boldsymbol{I}+\Delta  \hat{\mathbf{A}}\left({t}_k\right)\right) $$

where $\hat{\boldsymbol{A}}(t)$ is the matrix with entries ${\hat{A}}_{ij}(t)$, ***I*** is the identity matrix and $\Delta \hat{A}(t)$ are the difference in $\hat{A}$ between *t* and the time just prior to *t*

17 $$ \Delta {\hat{\mathrm{A}}}_{ij}(t)=\frac{number\ of\ observed\ i\to j\  transitions\  at\ t}{number\ of\ in dividuals\ \mathrm{a}t\  risk\ in\ state\ i\  just\ prior\ to\ t} $$

For example, the transition rate between the two states (1: Non-MV, 2: MV) is calculated by substituting 1 for *i* and 2 for *j* (i.e. Non-MV→MV) or substituting 2 for *i* and 1 for *j* (i.e. MV→Non-MV) into eq. (17). Eq. (17) is used to calculate the state occupation probabilities in eq. (16).

Beyersmann and Putter [12] describe how to estimate the sojourn times spent in State 1 and State 2 from the state occupation probabilities. This approach is also based on the Aalen Johansen estimators and implemented within the R-function *ELOS* in the *mstate* package. Confidence intervals can be obtained via bootstrapping.

In addition to describing the risk of death, giving the chances to be discharged, and quantifying hospital capacities (length of ICU stay, duration of mechanical ventilation), cause-specific hazard regression models can be used to study the potential impact of factors on each of the transition hazards.

For a more thorough treatment of the theoretical background of the paper, see Additional file 12.

### Data examples

#### Example 1: Case series of critically-ill COVID-19 patients in Seattle, USA

In the first example, we reconstructed the patients in the case series from Bhatraju et al. [8] by extracting the data depicted in a figure in their paper. The study included 24 laboratory-confirmed COVID-19 patients admitted to ICUs in the surrounding area of Seattle, USA. The paper provides individual patient information including treatment with invasive mechanical ventilation times, as well as final outcomes (discharged alive, dead). Periods of acute care were also provided but due to the small size of the sample, we dichotomized the patients into two states: “Non-MV” (ICU without MV and acute care) and “MV” (ICU with MV). This dichotomization matches the model presented in Fig. 1. Maximum follow-up was 31 days with each patient having at least 14 days of follow-up. At admission, 13 (54%) patients were Non-MV while 11 (46%) were MV. Seven patients were censored. Table 1 shows a portion of the data set extracted from the published figure and adapted to the model in Fig. 1. For example, patient with id 1 started Non-MV (‘from’ = 1) at ICU admission (‘entry’ = 0) and transitioned into MV (‘to’ = 2) on day 5 (‘exit’ =5). Patient 1 then moved back into Non-MV on day 16, before being censored on day 25 (‘to’ = 0). ID 2 died (‘to’ = 4) on day 1. The patient with ID 3 started MV, transitioned into Non-MV on day 12, and then is discharged (‘to’ = 3) on day 15. Data is required to be put into this form for the functions that are provided. The format can be easily adjusted to take into account baseline and time-dependent covariates. The entire data set for example 1 is provided in Additional file 2.

**Table 1:** Example structure of data set. Portion of data set from example 1 as extracted from Bhatraju et al. [8]. Full data set provided in Additional file 2. id: patient id, from: state entered at time ‘entry’, to: state entered at time ‘exit’, entry: time of entry into state ‘from’, exit: time of entry into state ‘to’

id	from	to	entry	exit
1	1	2	0	5
1	2	1	5	16
1	1	0	16	25
2	1	4	0	1
3	2	1	0	12
3	1	3	12	15

#### Example 2: Cohort study of patients with severe COVID-19 and treated with compassionate-use Remdesivir

Our second data example of COVID-19 patients is a reconstruction of the study population from Grein et al. [9]. The study included patients with severe COVID-19 that were treated with Remdesivir. Inclusion criteria were confirmed SARS-CoV-2 infection and an oxygen saturation of ≤94% or oxygen support. Follow-up was 28 days. Missing data regarding level of oxygen support was imputed by the method of last observation carried forward (LOCF). In this study we have detailed information not only on episodes of MV but also on other forms of intubation. To match the model shown in Fig. 1, we again dichotomized the patients into two groups: “Non-MV” (noninvasive positive pressure ventilation, nasal high-flow oxygen therapy, low-flow oxygen, ambient air) and “MV” (extracorporeal membrane oxygenation and invasive mechanical ventilation). At admission, 19 (36%) patients were non-MV while 34 (64%) were MV. Twenty-one patients were censored.

The data set for example 2 is provided in Additional file 7.

## Results

### Example 1

**Fig. 2:**
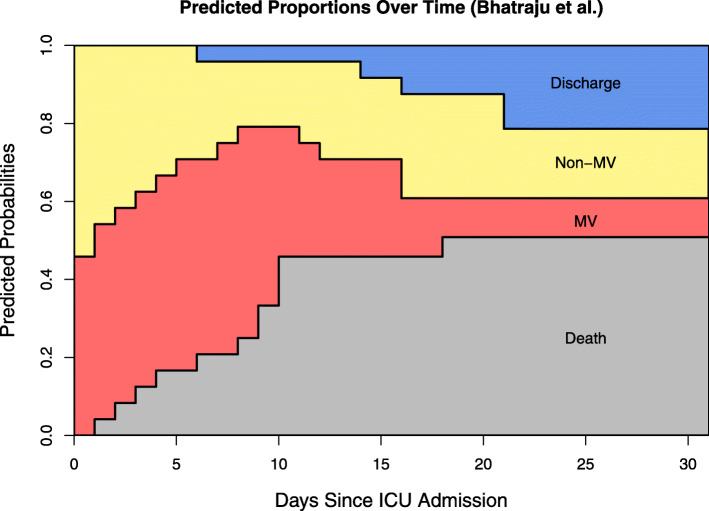
Example 1 Plot. Stacked probability plot for the data from example 1 [8] using the model in Fig. 1. Non-MV: intensive care unit without mechanical ventilation and acute care, MV: intensive care unit with mechanical ventilation

Predictions of the expected length of stay for patients in this cohort are shown in Table 2. For example, a patient starting unventilated at the beginning of his/her ICU stay had a much shorter expected duration of ventilation (4.34 days) than a patient already ventilated at ICU admission (12.25 days). Using the initial distribution, the weighted average of the expected durations in each state determined the expected total ICU time (15.05 days). This information is vital for advance planning of both ventilation and ICU capacities. The same is true for Fig. 2: we multiplied the transition matrix (eq. (16)) by the initial distribution to produce the stacked probability plot that illustrates the predicted proportions of the states throughout the entire follow up. At day 21 after ICU admission, for instance, a predicted 21% of patients are already discharged, 18% are not invasively ventilated, 10% need invasive mechanical ventilation, and mortality is predicted at 51%. With respect to the relatively high level of predicted mortality based on this cohort, it should be noted that 4 deceased patients had do-not-resuscitate orders in place before their admission. R code to reproduce the analysis for example 1 is provided in Additional file 5.

**Table 2:** Example 1 Results. Predicted sojourn times and mortality for patients in data example from Bhatraju et al. [8] at 28 days of follow-up. Start: time of ICU admission, Non-MV: ICU without MV and acute care, MV: ICU with MV, (): 95% confidence interval for duration estimates, (): standard error for risk estimates

	24 critically-ill COVID-19 patients in Seattle, USA (Bhatraju et al.), results at day 28			
	Non-MV Duration in Days	MV Duration in Days	Total Length of ICU Stay in Days	Death Risk
Start Non-MV	9.82 (5.84, 14.42)	4.34 (1.65, 7.7)	14.16 (7.49, 22.12)	47.8% (10.5)
Start MV	3.84 (1.12, 7.44)	12.25 (9.00, 16.03)	16.09 (10.12, 23.47)	54.4% (10.7)
Full Cohort	7.08 (4.00, 10.48)	7.97 (5.29, 11.18)	15.05 (9.29, 21.66)	50.8% (10.6)

### Example 2

**Fig. 3:**
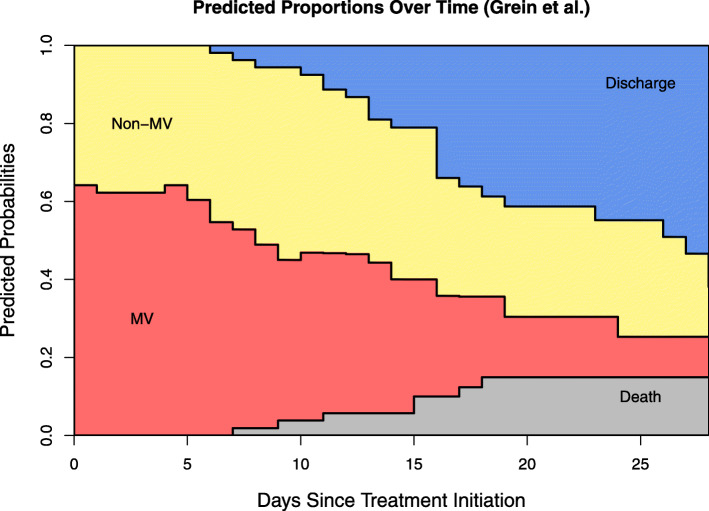
Example 2 Plot. Stacked probability plot for the data from example 2 [9] using the model in Fig. 1. Non-MV: noninvasive positive pressure ventilation, nasal high-flow oxygen therapy, low-flow oxygen, and ambient air. MV: extracorporeal membrane oxygenation and invasive mechanical ventilation

**Fig. 4:**
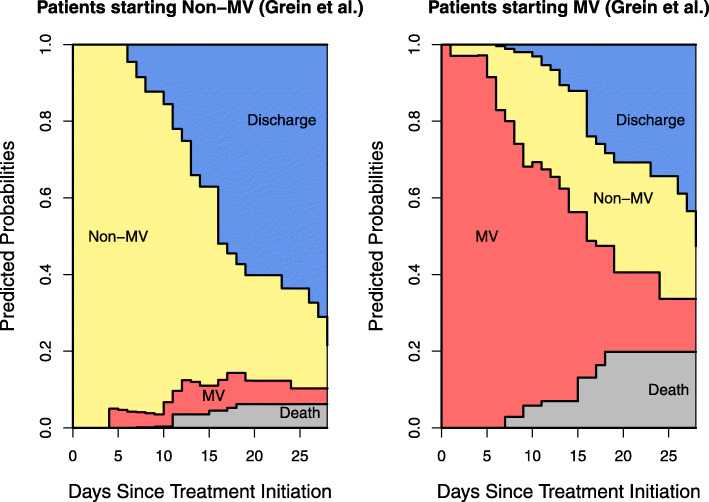
Example 2 Plot, Non-Ventilated vs. Ventilated on Day 0. Stacked probability plots for the data from example 2 [9] using the model in Fig. 1. Plots illustrate the clinical progression of patients who were not invasively ventilated at treatment initiation (left plot) and those who were (right plot). Non-MV: noninvasive positive pressure ventilation, nasal high-flow oxygen therapy, low-flow oxygen, and ambient air. MV: extracorporeal membrane oxygenation and invasive mechanical ventilation

The expected sojourn times and lengths of stay for this data are presented in Table 3. Figure 3 provides a visualization of the clinical course of the full cohort for example 2. Similar to example 1, patients initially MV had a longer expected ICU stay (20.71 vs. 17.67 days) at 28 days. Figure 4 sheds light onto this finding by comparing the clinical progression for patients starting in the two initial states. The increased size of the sample in example 2 allows such visual comparisons to be made. At 21 days of follow-up, patients starting in Non-MV had a higher probability of being discharged alive (60% vs. 31%) and a lower probability of dying (6% vs. 20%). ICU duration is shortened by a higher death probability in initially MV patients and a higher discharge probability for initially non-MV patients. This underlines the influence these competing events have on the lengths of stay. Figure 4 visually illustrates the marked difference in the progression of the hospital stay of patients in these two groups. It indicates that the ventilator demands of patients who are initially admitted non-ventilated are different from those who are ventilated at admission. R code to reproduce the analysis for example 2 is provided in Additional file 8.

**Table 3:** Example 2 Results. Predicted sojourn times and mortality for patients in data example from Grein et al. [9] at 28 days of follow-up. Start: time of treatment initiation, Non-MV: noninvasive positive pressure ventilation, nasal high-flow oxygen therapy, low-flow oxygen, and ambient air, MV: ECMO and MV (): 95% confidence interval for duration estimates, (): standard error for risk estimates

	53 patients with severe COVID-19 treated with Remdesivir (Grein et al.), results at day 28			
	Non-MV Duration in Days	MV Duration in Days	Total Length of ICU Stay in Days	Death Risk
Start Non-MV	16.26 (13.87, 18.56)	1.41 (0.27, 2.96)	17.67 (14.14, 21.52)	6.2% (3.3)
Start MV	6.14 (3.86, 8.41)	14.57 (11.99, 17.31)	20.71 (15.85, 25.72)	19.8% (6.4)
Full Cohort	9.77 (7.76, 11.81)	9.85 (7.68, 12.14)	19.62 (15.44, 23.95)	15.0% (5.3)

## Discussion

We have demonstrated how researchers can model the hospital stays of COVID-19 patients to determine the expected duration of mechanical ventilation, expected overall ICU stay, and patients’ predicted clinical progression while avoiding common pitfalls and biases in modeling such settings. Given the need for reliable evidence, we believe that the application of multistate models to this kind of data represents the best way to generate an extensive amount of valid evidence from observational studies.

Although the limited number of patients in the two data examples made it difficult to identify predictors or analyze treatment effects, visualizations of the results provide easy-to-interpret and comprehensive information of the patients’ clinical courses. The results of the two re-analyses provide insights into time-dependent event-probabilities, while estimates regarding the conditional length of stay are of major interest for capacity planning. To maintain transparency and further help researchers, code in the programming language R and the data examples are provided in the additional files.

The model selected in Fig. 1 facilitated a harmonization of the two data examples. The data sets could have, in fact, been merged if not for differing time origins (time from ICU admission vs. time from Remdesivir treatment initiation). Nonetheless, the harmonization reveals the potential for use in metanalyses and systematic literature reviews. In contrast, the flexibility of the methodology is illustrated in the various models that could have been chosen for each of the data examples. Although we dichotomized the states into Non-MV and MV, example 1 also provided information on acute care while example 2 included information on 6 different levels of oxygen support. Additionally, a transition state of ‘ICU after invasive ventilation’ could be modeled to give further insights into the healthcare demands of ventilated COVID-19 patients. To demonstrate the flexibility of the methodology, an extended model analysis for example 2 is provided in Additional file 11. A researcher can adapt the choice of multistate model to the data or outcome of interest.

Since publication, several researchers [15] have pointed out biases in the analyses performed by Grein et al. The bias stems from the censoring of deceased patients, whose risk of improving is not similar to non-censored patients (i.e. informative censoring). This results in an overestimated cumulative incidence of clinical improvement. It should be noted that this bias would have been avoided with our proposed multistate methodology as death and discharge from the ICU are absorbing states from which patients are no longer at risk of clinical improvement.

In addition to the biases common to survival analysis already mentioned, Lipsitch et al. [16] review biases that can occur during the outbreak of both known and unknown infectious diseases. They describe a “survivorship bias” that can occur when many infected patients die before being hospitalized, thus implying a protective effect of hospitalization on mortality. This bias is related to “length bias” [6, 7] which can be addressed by incorporating left-truncation. Lipsitch et al. suggest comparing the risk of death among patients hospitalized and non-hospitalized on a specific day since a patient has become a case, and combining the estimates over several days. Here multistate methodology could be applied by modeling the transient states of “hospitalized” and “non-hospitalized” with a transition into “death” from both states. There are several advantages to this model. First, all patients can be included in one analysis through assignment into one of the initial states (“hospitalized” and “non-hospitalized). Similar to our comparison of stacked probability plots in Fig. 4, we could then compare the risk of death for these two groups. Second, the model accounts for the time-dependent nature of hospitalization by allowing for several admission and discharge transitions. The allowance of repeated transitions among initial states is an advantage over standard competing risk models. This further highlights the utility of the proposed methods in epidemic/pandemic settings.

Although the sizes of our two data samples are rather modest, the volume and availability of COVID-19 data is expanding. These methods applied to more detailed patient data could produce very informative plots for comparing age, gender, underlying health condition, or even different treatment arms [17]. This expanding capability to compare groups visually as data sets increase in size was demonstrated in Fig. 4, which was informative for the larger of our two real data sets. Furthermore, depictions like the second figures in Bhatraju et al. [8] and Grein et al. [9] provide an impression for the viewer when the sample sizes are small. However, such depictions are overwhelming and difficult to read with larger data sets. In contrast, the stacked probability plots incorporate all of the information in the aforementioned figures into one easy-to-view illustration regardless of the size of the sample. Naturally, the precision of the stacked probability plots increases with an expanding number of patients.

There are several limitations to our demonstration. First, we chose the model in Fig. 1 as it reflected the observed transitions recorded in the two data sets. The observations included patients who were discharged from the ICU directly from being mechanically ventilated; in other words without first being non-ventilated in the ICU. From a clinical standpoint, these observed transitions do not occur. Either the patients were extubated and remained in the ICU for a couple hours, or were transferred to another ICU unit. In either case, there may be reason to adjust the data set by censoring these observations. Second, the lengths of stay estimates do not distinguish between the final outcomes of discharge alive and death. While these estimates are relevant for planning capacities, they are less clinically relevant. An alternative would be to evaluate, for example, time alive without mechanical ventilation. Third, we performed LOCF on the example 2 data to handle periods of missing information on a patient’s level of oxygen support. While this simplified the analysis, it is likely that transitions between states occurred for longer periods of missing information.

A further strength of this methodology is that it allows for censoring, thus acknowledging active cases. It therefore lends itself to ongoing as well as completed studies. Considering the wealth of COVID-19-related research being produced currently, the multistate approach is an invaluable addition to a COVID-19 researcher’s toolkit.

## Conclusions

Applying multistate methodology to ICU settings with COVID-19 patients gives important insights into mechanical ventilation duration, length of ICU stay, and mortality. The visualization of these results, in the form of a stacked probability plot, is both easy-to-read and comprehensive. The approach also allows for clear comparisons among different baseline characteristics, and even treatment arms. The tools described here offer important aid to decision makers with regard to healthcare capacities.

## Supplementary information

**Additional file 1.** format: txt, title: README_Example1.txt, description: README file with instructions so that a researcher can reproduce the results for data example 1.

**Additional file 2.** format: csv, title: Example1_Data.csv, description: Data set for example 1.

**Additional file 3.** format: R, title: ext_mstate.R, description: R function to modify data frame for use in R package mstate.

**Additional file 4.** format: R, title: LOS_boot.R, description: R function to calculate bootstrap confidence intervals in R package mstate.

**Additional file 5.** format: R, title: Example1_Analysis.R, description: R code to reproduce the results for data example 1. Additional files 2, 3, and 4 must be in the R working directory with Additional file 5.

**Additional file 6.** format: txt, title: README_Example2.txt, description: README file with instructions so that a researcher can reproduce the results for data example 2.

**Additional file 7.** format: csv, title: Example2_Data.csv, description: Data set for data example 2.

**Additional file 8.** format: R, title: Example2_Analysis.R, description: R code to reproduce the results for data example 2. Additional files 3, 4, and 7 must be in R working directory with Additional file 8.

**Additional file 9.** format: txt, title: README_Example2_Extended.txt, description: README file with instructions so that a researcher can produce results for extended data example 2.

**Additional file 10.** format: csv, title: Example2_Extended_Data.csv, description: Data set for extended data example 2.

**Additional file 11.** format: R, title: Example2_Extended_Analysis.R, description: R code to produce the results for extended data example 2. Additional files 3, 4, and 10 must be in R working directory with Additional file 11.

**Additional file 12.** format: docx, title: Theoretical_Background.docx, description: Theoretical aspects of the analyses of the real data examples.

## Data Availability

All data generated or analyzed during this study are included in this published article (and its supplementary information files).

## Abbreviations

ECMO: Extracorporeal membrane oxygenation
ICU: Intensive Care Unit
LOCF: Last observation carried forward
MV: ICU with mechanical ventilation
Non-MV: ICU without mechanical ventilation
