# Supplementary material for: Joint analysis of duration of ventilation, length of intensive care, and mortality of COVID-19 patients: a multistate approach
Source: BMC Med Res Methodol. 2020 Aug 11;20:206. doi: 10.1186/s12874-020-01082-z (PMC7507941; doi:10.1186/s12874-020-01082-z)
Supplement: Supplementary file 12 — Additional file 12. format: docx, title: Theoretical_Background.docx, description: Theoretical aspects of the analyses of the real data examples. [file 12874_2020_1082_MOESM12_ESM.docx]

**THEORETICAL PART OF PAPER**

**1. Theoretical Aspects of State Occupation Times**

The theoretical approach in this paper is based on continuous-time Markov chains and their application in the framework of multistate models. The basic tool is given by product integration. This theory is already widely explored and used in many mathematical ﬁelds [7]. The main goal is to derive formulas for the average staying time, sometimes called occupation time, in a given state. Those quantities can be explicitly calculated using transition probabilities which will be explained using basic notation.

In the following we consider a time-inhomogeneous Markov chain given by $\{X\left( t \right), t \geq0\}$ with ﬁnite state space $S=\{1, 2, \ldots, N\}$. As usual, we assume that the process has right-continuous sample paths which are additionally piecewise constant from a regularity point of view. To clarifyﬁy this property, note that if the process $X$ is moving from some state *l* to state *m*, where we assume that $l\neq m$, at some time *T*, then

$X\left( T \right)=m$ and $X\left( T^{-} \right)=l$.

Clearly, $X(T^{-})$ refers to the state which was occupied just before the actual time point $T$. Additionally, it’s a reasonable assumption to say that on a given ﬁnite time interval, let’s say $[0, \tau]$, we only have ﬁnitely many transitions between the states in *S*. Talking about Markov models, we brieﬂy recall the actual Markov property which will play a crucial role and simpliﬁes many calculations. The Markov property, sometimes called memorylessness [6], is given by

$P(X(t)=m| X(s)=l, History of the process)=P(X(t)=m| X(s)=l)$,

where ’History of the process’ is the actual past the process has witnessed. In the end, the Markov property simply states that the conditional probability of future states, e.g. $X(t)=m$, of the process only depends on the present state and not on the past events. In mathematical terms this is done using a *σ*-algebra, which is dependent on the process and covariates, for more details we refer to [2] and [5].

The actual transition probabilities between two states, say $(l,m)\in S^{2}$with $l\neq m$, are given by

$P_{lm}(s,t) :=P(X(t)=m| X(s)=l), \mathrm{for} s\leq t$*.*

Using this notation it is useful to deﬁne the transition probability matrix which is simply given by

$$\mathbf{P}(s,t) = \left( P_{lm}\left( s,t \right) \right)_{l,m} \mathrm{for} l,m \in S.$$

Using a special property, called a halfgroup property [6], we can multiply the initial distribution $(P(X(0)=1),P(X(0)=2),... , P(X(0)=N))$with the matrix $\mathbf{P}(s,t)$to actually derive the state occupation probabilities at some time t given by the vector

(1)

$(P(X(t)=0),P(X(t)=1),...,P(X(t)=N))$.

**Remark and Example.** An example for an initial distribution vector would be$(1,0,...,0) \in\mathbb{R}^{N}$. Roughly translated, this means that all individuals start in state 1. Keep in mind that for a matrix multiplication resulting in equation (1) one needs to consider the correct order of multiplication terms.

Upon using the components in the state occupation probability vector we can calculate the expected time in some state $m$. This quantity of interest is given by

$$E\text{m}=\int_{0}^{\infty} P\left( X\left( u \right)=m \right)du,$$

see [3]. Certainly, this is not the only way to deﬁne an occupation time. Another method is to recall a proper random variable, which is simply given by the minimal time the process $X$ is leaving state m. Hence, the state occupation time is given as the expected value of such a random variable. If we assume that we are dealing with a homogeneous continuous-time Markov chain, this random variable is exponentially distributed, see [6].

Clearly, in reality it is not possible to have an inﬁnite observation time, hence another quantity needs to be addressed. Note that in many cases we only have a ﬁxed time of observation, let say $\tau< \infty$. Hence we observe the status of an individual only in the ﬁxed interval given by$[0,\tau]$. In such a case we can still calculate the expected occupation time in a given state, conditioned on simply observing everything in the interval $[0,\tau]$. This quantity is given by

$$E_{m}^{\tau}=\int_{0}^{\tau} P\left( X\left( u \right)=m \right)du,$$

see [3].

**Remark.** The values $E_{m}$ and $E_{m}^{\tau}$ also have a very nice geometric interpretation. We note that both quantities actually are given by integration of a certain function, in our case the probability $P(X\left( u \right)=m)$. Hence we can see that the state expected state occupation times are actually given by the area.

In very simple scenarios it might be possible that those quantities behave very tame and can easily be calculated. A ﬁrst example is to use a homogeneous continuous-time Markov chain, which has constant hazard rates [5]. Additionally, in [3] an obvious connection between the transition probability matrix $\boldsymbol{P}(s,t)$ and the cumulative transition hazards $A_{lm}(s)$ is explored. Simply put, taking a partition of the time interval $[s,t]$ given by $s=t_{0}<t_{1}<t_{2}< ... <t_{L}=t$ we get

$$\mathbf{P}\boldsymbol{(}s,t\boldsymbol{)\approx}\prod_{l=1}^{L} \left( \boldsymbol{I}+\Delta\boldsymbol{A}\left( t_{l} \right) \right).$$

Roughly spoken, taking a ﬁner and ﬁner partition (see [7]), which ultimately leads to a limit given by a product integral, we see that

(2)

$$\mathbf{P}\left( s,t \right)= \prod_{u\in(s,t]} \left( \mathbf{I}+d\mathbf{A}\left( u \right) \right),$$

check [3] for more details and references.

**Remark.** For even more details on such operators, the interested reader may check out [4]. And for a technically more involved article, [1] is written in a clear, precise and understandable way.

Relation 2 readily prepares for a natural estimate. An estimate for the state occupation time E_m_ is simply given by

$$\hat{E_{m}}=\int_{0}^{\infty} \hat{P}\left( X\left( u \right)=m \right)du,$$

where $\hat{P}\left( X\left( u \right)=m \right)$ can be calculated from estimates of the initial probability distribution already discussed. Note that estimate in the case of a ﬁxed observation interval can be calculated in a similar fashion, e.g. for $E_{m}^{\tau}$.

Very often it is the case that the cumulative transition hazards, i.e. $A_{lm}$, are actually estimated using step functions. A prominent non-parametric method is the Nelson-Aalen estimator and for a semi-parametric method the Cox model comes to mind. In such a case the quantity $\hat{P}\left( X\left( u \right)=m \right)$ is actually constant with respect to time $u$. As a simple example, consider that

$$\hat{P}\left( X\left( u \right)=m \right)=\hat{p_{l}} on the interval \left[ q_{l} \right.-1,q_{l}) \mathrm{for}l=1,\ldots,L,$$

note that $q_{0}=0$ and $q_{L}=\infty.$ Clearly, the ﬁner the partition (e.g. the bigger $L$), the better the result in the end. In this simple case we readily check that

$$\hat{E_{m}}=\sum_{l=1}^{L} (q_{l}-q_{l-1}) \hat{p_{l}}.$$

As a simple remark, we note that if $\hat{p_{L}}$ is not $0$ then the sum will not converge. This means that $\hat{E_{m}}=\infty$. This is easily seen to happen in an absorbing state. Again, those relations are properly explored in more detail in [3].

**Remark.** For the quantity $\hat{E_{m}^{\tau}}$ we need to make a little change. In that case we simply let $q_{L}=\tau$ and calculate the quantity as above.

**References**

1. Odd O. Aalen, Per Kragh Andersen, Ørnulf Borgan, Richard D. Gill and Niels Keiding, History of applications of martingales in survival analysis, 2010, arXiv:1003.0188 [stat.ME].

2. P. K. Andersen, O. Borgan, R. D. Gill and N. Keiding, Statistical Models Based on Counting Processes, Springer, Springer Series in Statistics, 1993.

3. J. Beyersmann and H. Putter, A note on computing average state occupation times, Demographic Research, vol. 30, article 62, 2014.

4. O. Borgan, Three contributions to the Encyclopedia of Biostatistics: The Nelson-Aalen, KaplanMeier, and Aalen-Johansen, University of Oslo, DUO Research Archive.

5. R. J. Cook and J. F. Lawless, Multistate Models for the Analysis of Life History Data, Chapman & Hall CRC Press, Monographs on Statistics and Applied Probability, 2018.

6. W. Feller, Introduction to Probability Theory and Its Applications, Vol II (2nd edition), Wiley, 1971.

7. A. Slavik, Product integration, its history and applications, Matfyzpress, Prague, 2007.
